# Review: Application of Protein-Based Raw Materials in Health Foods in China

**DOI:** 10.3390/foods14010020

**Published:** 2024-12-25

**Authors:** Hao Duan, Gaigai Liu, Jiaqi Liu, Zhuoye Wang, Shuyuan Bao, Xinyue Chang, Wenjie Yan

**Affiliations:** 1 College of Biochemical Engineering, Beijing Union University, Beijing 100023, China; dhuanao@163.com (H.D.); lgg010403@163.com (G.L.); liujiaqi0711@foxmail.com (J.L.); cxy562380436@163.com (X.C.); 2 Beijing Key Laboratory of Bioactive Substances and Functional Food, Beijing Union University, Beijing 100023, China; 3 Xiangya School of Public Health, Central South University, Changsha 410083, China; 2022050352049@buu.edu.cn (Z.W.); 8304230626@csu.edu.cn (S.B.)

**Keywords:** protein-based ingredients, health food, functionality, application status, compliance

## Abstract

Raw protein materials are beneficial for human health, so they are being increasingly used in health foods. In recent years, there has been more and more research on and applications of raw protein materials, but few teams have conducted a detailed review of the application status of raw protein materials in China’s health foods, the basis for their compliance and use, and the research on their health care functions. Therefore, this review evaluates the application of animal and plant proteins in China’s health foods, the impact of animal and plant proteins on human health, and future research recommendations for animal and plant proteins. This review analyzes and discusses the data on approved health foods that have been verified to contain raw protein materials (mainly including the number of protein health foods approved over the years, the classification of raw protein materials and types of relevant regulations, the analysis of the frequency of use of raw protein materials, and the functions of approved health foods). Through this process, the application of raw protein materials in health foods in China is systematically reviewed. In short, through data analysis, this study found that in 1996~2024, a total of 1142 health foods containing raw protein materials were approved in China, which are mainly divided into animal proteins, vegetable proteins, microbial proteins, and peptide raw materials, and peptide raw materials comprise the majority. The compliance applications of these ingredients are mainly related to China’s five categories of food regulations. The results show the following for health foods containing raw protein materials: in terms of the dosage form, they are mainly solid preparations; according to their functional claims, they mainly help to enhance immunity, help improve bone density, help improve skin moisture, and relieve physical fatigue; and in the application of raw materials, it is found that the use of raw materials such as casein phosphopeptide, soybean protein isolate, whey protein, collagen, spirulina, and other raw materials in products is relatively high. Finally, based on these studies, this paper discusses suggestions for raw protein materials in the future development of health food in China and also discusses the limitations of the current research in this review.

## 1. Introduction

Raw protein materials are divided into proteins and peptides, the sources of which can be roughly divided into animals, plants, and microorganisms. Protein is one of the essential macronutrients for human beings; it is a macromolecular substance with a certain spatial structure formed by amino acids connected with peptide bonds [1]. Amino acids of different types, in different quantities, and with different sequences and spatial structures constitute a wide variety of proteins, which provide the support necessary for the body’s immunity and cardiovascular health [2,3]. Peptides are formed by the dehydration of amino acids or are small molecular compounds obtained from natural proteins via enzymatic digestion or hydrolysis; they possess a wide range of biological activities and are easily absorbed by the stomach, intestines, blood vessels, and skin [4,5,6] and thus have a wide range of applications in cosmetic and gastrointestinal health [7].

In today’s era of pursuing a healthy life and improving quality of life, the health food industry is booming. As an indispensable and important material basis for human life activities, protein plays a key role in maintaining normal physiological functions, promoting growth and development, repairing tissue damage, and regulating immunity [1]. Globally, with the continuous awakening of consumers’ health awareness and the growing demand for nutritional supplements, raw protein materials have gradually become one of the core ingredients in the field of health food. Proteins are widely used in various health foods in rich and diverse forms, ranging from traditional animal and plant proteins, such as whey protein and soy protein, to emerging ones, such as collagen, marine proteins, and other protein sources. These protein-based ingredients not only give specific nutritional value and functional properties to the product but also meet the individual needs of different people with specific health conditions, such as helping to enhance immunity, improve bone density, improve skin hydration, relieve physical fatigue, etc. It is of great significance to deeply explore the application of raw protein materials in China’s health foods to promote innovation and development in the health food industry, improve product quality and efficacy, and better serve the health demands of consumers. This review evaluates the application of animal and plant proteins in China’s health food, the impact of animal and plant proteins on human health, and future research recommendations for animal and plant proteins. This review analyzes and discusses the data on approved health foods that have been verified to contain raw protein materials (mainly including the number of protein health foods approved over the years, the classification of raw protein materials and types of relevant regulations, the analysis of the frequency of the use of raw protein materials, and the functions of approved health foods). Through this process, the application of protein raw materials in health foods in China is systematically reviewed. At the same time, this paper analyzes the application and formula compatibility of raw protein materials in health foods with four functions: helping to enhance immunity, helping to improve bone density, helping to improve skin hydration, and relieving physical fatigue. This review provides a reference for the development, functional research, and compliance declaration of health food containing raw protein materials.

## 2. Method

The first step is to collect, screen (including exclusion and inclusion), and statistically analyze products containing raw protein materials in Chinese health foods; the second step is to perform a narrative analysis, which involves literature collection, screening (including exclusion and inclusion), and categorization.

In the first step, product-information-related data (product name, product formula composition, product quantity, and product dosage form) on China’s health foods come from the National Special Food Information Query Platform of the State Administration for Market Regulation (SAMR). The website address is http://ypzsx.gsxt.gov.cn/specialfood/ (accessed on 9 June 2024). “protein”, “peptide”, “enzyme”, and other keywords were used to retrieve the product. The retrieved data were downloaded, the products with missing key information such as product name and product formula composition were eliminated, and the data with perfect product information were included in the final analysis. A total of 1142 effective product data were obtained, and then the data analysis of product name, product formula composition, product quantity, and product dosage form was carried out. The basis for the use of these data (regulations) was approvals over the years. Data such as the type of raw materials and the frequency of use of raw materials were analyzed and discussed.

The second part of the narrative examination was carried out in three steps: conducting the search, examining the abstract and full text, and discussing the results. To this end, PubMed, Scopus, Science Direct, Web of Science, Science Direct, CNKI, and Google Scholar databases were searched as the review progressed to identify relevant studies. The final search was conducted in July 2024 and included international English articles, online reports, and e-books. Keywords “animal protein”, “plant protein”, and “protein” were used in conjunction with other terms such as words such as bone density, immunity, skin, physical fatigue, etc. Once we completed the search, we read the summary to make sure the study was relevant to the topic of interest. We removed all duplicate articles and reviewed the abstracts of the remaining articles to ensure they meet the review inclusion criteria. Eligibility criteria are divided into three dimensions (animal protein, plant protein, and the role of animal and plant proteins on human health). Therefore, studies of interest focusing on these three dimensions were summarized and synthesized to integrate narrative reviews.

**Figure 1:**
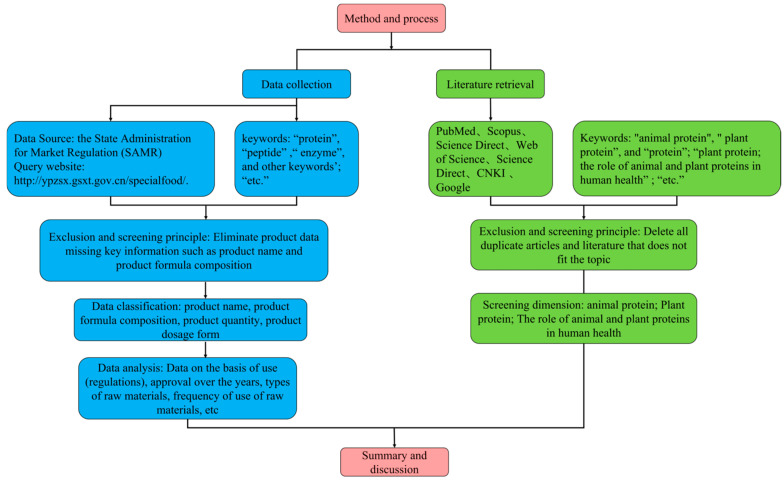
Flowchart of the method.

As this is a narrative review, there is no need to record a literature search on a specific platform. The flowchart of the method is shown in Figure 1.

## 3. The Basis for the Use of Protein-Based Raw Materials in Health Food in China

In China, the National Health Commission of the People’s Republic of China issued GB 16740-2014, “National Food Safety Standard Health Food”, which defines health food as “food that claims and has specific health functions or is to supplement vitamins and minerals”. That is, it is suitable for specific groups of people to eat, can regulate the body’s functions, is not to treat diseases, and does not produce any acute, subacute, or chronic harm to the human body. Before the listing of Chinese health foods, they need to submit relevant materials for health food registration declaration or filing application to the Center for Food Evaluation and State Administration for Market Regulation (SAMR). After they have been strictly reviewed and approved, they can enter the market and be provided to consumers; that is, the registration and filing classification management system is implemented.

Data collection from the special food information query platform (http://ypzsx.gsxt.gov.cn/specialfood/#/food, accessed on 9 June 2024) of the State Administration for Market Regulation obtained 1142 products containing raw protein materials, and the basis for the use of these raw materials can be roughly divided into 5 categories in the “Catalogue of Health Food Raw Materials” issued by the State Administration for Market Regulation, food nutrition enhancers, and health food excipients. There are five categories of new food raw materials and ordinary food raw materials, and the attributes of these raw materials are found in all foods. Clear literature research data should be provided when used as functional raw food materials. Table 1 summarizes the basis and details of the use of healthful raw protein food materials.

### 3.1. Health Food Raw Materials

SAMR Document No. 22 of 2023 included soy isolate protein and whey protein in the catalog of health food ingredients, with the approved health care function of immunity enhancement, and the dosage ranges of both were 6~25 g of protein. Meanwhile, the document “Health Food Ingredients Catalog Nutrient Supplements (2023 Edition)” clarifies that selenoprotein and casein phosphorpeptide + calcium can be used in health food nutrient supplements. In addition, the raw protein materials included in the catalog of health food ingredients also include spirulina, the source of which is blunt-topped spirulina (Arthrospira platensis) and extremely large spirulina (Arthrospira maxima) approved, whose function is also to enhance immunity. Its dosage is 3~4 g/d. At the same time, these two types of spirulina were explicitly declared as general food in Document No. 17 of 2004, so they can also be used as general food.

### 3.2. Health Food Accessories and Food Fortification

Currently, the protein-based raw materials included in health food excipients are sodium caseinate, the implementation standard of which refers to GB 1886.212 “National Standard for Food Safety Food Additives Sodium Caseinate (also known as sodium caseinate)”. As well as ice structural protein, its implementation standard reference GB 1886.299-2018 includes “Food Safety National Standard Food Additives Ice Structural Protein”.

Three types of protein ingredients belong to food fortification: selenoproteins, casein phosphopeptides, and lactoferrin.

### 3.3. New Food Ingredients

New food ingredients refer to animals, plants, microorganisms (or ingredients isolated from these three), food ingredients whose original structure has been changed, or other newly developed food ingredients that are not traditionally consumed in China [8]. There are four categories of review and approval of new food ingredients: “recommended for approval”, “terminated for review”, “recommended for disapproval”, and “deferred for reconsideration” [9]. Currently, there are 10 new protein-based food ingredients approved by the Health and Welfare Commission: hydrolyzed egg yolk powder, colostrum alkaline protein, milk alkaline protein, geoduck protein (*Lumbricus protein*), protein nucleated chlorella (*Chlorella pyrenoidosa*), nudibranch (*Euglena gracilis*), globular candida (Gossypium) (*Nostoc sphaeroides*), micrococcus anthropomorphus (*Nannochloropsis gaditana*), Cladosiphon rhinoceros (*Chlamydomonas reinhardtii*), and yeast protein.

### 3.4. General Food Ingredients

Common food ingredients have a long history of consumption and are extremely safe and, therefore, can be used as health food ingredients [10,11]. There are three main categories: one is ordinary food, such as food processing with vegetable protein, casein, collagen peptides, etc., where the use of these raw materials needs to comply with the corresponding national standards; the second is the national announcement or letter of reply to the raw materials explicitly for the ordinary food ingredients; the third is the termination of the review of the new food ingredients judged to be ordinary food or food with ordinary food, which has to be referred to as the equivalent of the raw materials.

## 4. Application Status of Protein-Based Raw Materials in Health Food in China

### 4.1. Status of Approval of Protein-Based Ingredients in Health Food in China

#### 4.1.1. Analysis of Ratifications over the Years

**Figure 2:**
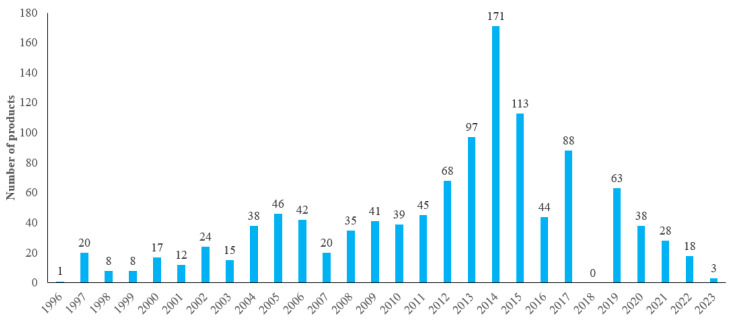
Protein-based raw materials approved for health food in China.

As can be seen in Figure 2, as of January 2024, data retrieved from the SAMR National Special Food Information Query Platform showed that a total of 1142 health food products containing protein-based ingredients were approved. Among them, the use of protein-based raw materials in health food was 0 in 2018, and it is presumed that the reason for this situation is related to the abolition of the Technical Code for the Inspection and Evaluation of Health Food (2003 version) in the “Decision of the Health Commission on Announcing the Invalidation of the Third Batch of Commission Documents (NHB [2018] No. 15)” in July 2018.

#### 4.1.2. Analysis of the Application of Protein-Based Raw Materials

Based on the statistical data (refer to Table 2), there are 56 peptides, 43 animal proteins, 9 plant proteins, and 1 microbial protein among the raw protein materials used in health food. Among them, casein phosphopeptide, marine fish skin collagen oligopeptide, soy peptide, whey protein peptide, marine fish bone collagen oligopeptide, collagen peptide, marine fish oligopeptide, soybean oligopeptide, and fish collagen peptide are the most widely used; hydrolyzed collagen, sheep placenta hydrolysate, and hydrolyzed proteins are the most widely used in hydrolyzed peptides, and enzymatic bone calcium powder and enzymatic ovalbumin are the most widely used in enzymatically hydrolyzed peptides. Among the animal proteins, whey protein, collagen, whey protein concentrate, collagen, lactoferrin, fish collagen, fish protein, colostrum alkaline protein, casein, ovalbumin, and egg white protein are the most widely used. Soybean isolate protein, soy protein, protein nucleus chlorella, pea protein, wheat protein, vegetable protein, barley protein, gluten, soy protein fiber, and selenium protein are the most widely used in plant proteins. Only one raw material, spirulina, has been used as a microbial protein. It is speculated that the reason for the greater application of peptide raw materials may be due to the better absorption of animal protein and peptides and the clearer efficacy.

Judging from the data obtained in the system, there are a total of 56 kinds of raw materials with a low frequency of use, and most of the raw materials are ordinary food raw materials or edible animal or plant proteins as raw materials. The substances made via the enzymatic hydrolysis of food enzyme preparations are allowed to be used in the “Standard for the Use of Food Additives” (GB2760-2011). Only four of the raw materials are derived from plant proteins, and the rest are derived from common food ingredients and edible animal proteins. In general, the types of animal and plant raw protein materials are abundant, and their application is more extensive than that of plant protein. Thus, it is necessary to dig deeper into the health care effects of animal raw protein materials and increase the research on the difference between plant proteins to improve the bioavailability of plant protein.

### 4.2. Application of Protein-Based Raw Materials to Support Functional Claims in Health Foods in China

**Figure 3:**
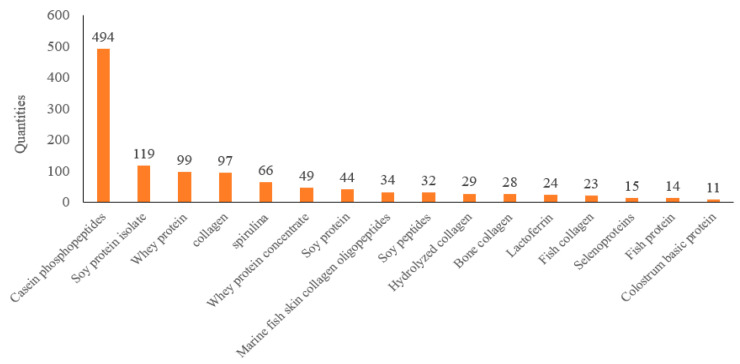
Protein-based ingredients used ≥10 times in approved products.

**Figure 4:**
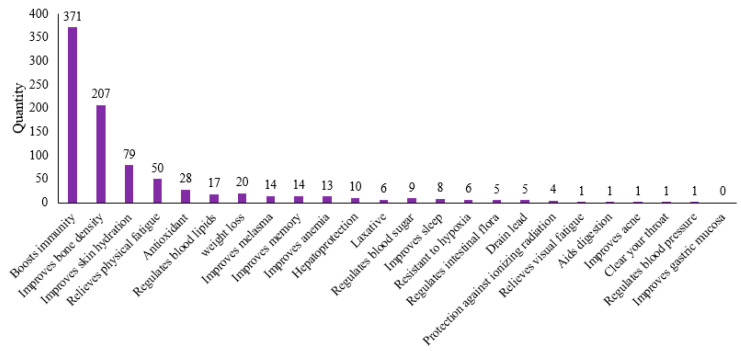
Application of protein-based raw materials for functional claims in health foods in China. Note: There are also 366 products claiming to supplement calcium, zinc, multivitamins, etc., which are not included in the statistics.

Figure 3 presents the usage frequency of various protein-based raw materials. In Figure 4, the distribution of functional claims for protein-containing raw materials in Chinese health foods is depicted. Among them, the number of products claiming to enhance immunity and improve bone density is conspicuously higher than that of other functions.

### 4.3. The Proportion of Protein-Based Raw Material Dosage Forms in China’s Health Food Products

**Figure 5:**
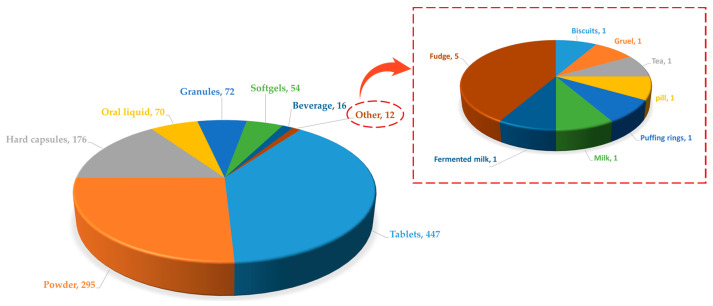
Application of protein-based raw materials in dosage forms of health food.

As can be seen in Figure 5, the current dosage forms of health food containing protein-based raw materials are mostly solid formulations, while liquid formulations account for less than 10%, which may be related to the poorer stabilization of liquid formulations. In general, when conditions such as pH, temperature, metal ions, and light in a liquid formulation change, the conformation of the protein may change, and biological activity may be lost [12]. The complexity of the environment is further enhanced when multiple raw materials are combined in a product, resulting in less stable liquid formulations with reduced absorption and utilization. Secondly, some orally consumed proteins and peptides have undesirable flavors, such as bitter, astringent, soybean, etc. In addition, the raw materials are easily hygroscopic, which makes it necessary to add a variety of flavor enhancers to liquid formulations to improve this effect. The solid preparation processing steps are simpler, and the processing cost is cheaper compared to the liquid preparation. Due to the better stability, the package material selection is also enhanced, the dose is clear, and the consumption is also more convenient. Solid dosage forms can also mask undesirable odors from raw materials through simple process steps, such as coating the outside of tablets and capsule shells for capsules, and such dosage forms also help protect against moisture.

The dosage form design needs to ensure that the product can fully utilize its health benefits, followed by a good taste. Therefore, the study of oral bioavailability and flavor of proteinaceous ingredients is crucial. Generally, suitable carriers will be chosen to encapsulate and deliver protein and peptide raw materials to avoid excessive loss under unfavorable conditions and mask their undesirable flavors, fully retaining their active ingredients so that they can be released and further absorbed in the small intestine to exert their proper health effects. In the current study, liposomes, nanoemulsions, and hydrogels are mostly used as delivery systems for raw protein materials [13]. It was found that the soy peptide–chitosan nanoparticle system based on tamarind polysaccharide and carboxymethyl cellulose can improve the release behavior of soy peptides in the oral cavity with good solubility, easy erosion by saliva, and good bioaccessibility of the oral mucosal route of administration to avoid the metabolism of the intestinal tract and the liver, which can control the release and absorption of peptides in the oral cavity [14]. In addition, carboxymethylcellulose/poly (vinyl alcohol) hydrogels loaded with soy peptides were prepared using the freeze-thawing method, which was able to overcome the barrier in the simulated gastric fluid and further release the soy peptides in the simulated intestinal fluid, effectively improving their absorption [15]. Although casein phosphopeptides (CPPs) can enhance calcium absorption by inhibiting the formation of insoluble calcium deposits, the peptides are susceptible to enzymatic degradation in the stomach before entry into the intestinal tract [16]. Therefore, some studies have used chitosan oligosaccharide core–shell particles of calcium (Ca) conjugated to CPP for calcium delivery, and the results of these studies showed that the particles of CPP-Ca resulted in a controlled release of calcium and sustained calcium absorption, significantly enhancing the bioavailability of Ca [17]. The use of a strong cross-linking agent, phytic acid, to encapsulate proteins in chitosan microcapsules was able to improve their encapsulation efficiency, digestive stability, control of gastrointestinal release, and oral bioavailability, and the results of the encapsulation system evaluated using an animal model of skin photo-aging showed a significant increase in the anti-dermal photodamageable and antioxidant effects of the encapsulation system [18]. Therefore, in future research, on the one hand, the comprehensive properties comprising structural and functional attributes of raw protein materials can be extensively studied and explored so that the preparation and embedding can be more stable. On the other hand, the research and preparation of delivery systems can be increased, the bioavailability of raw protein materials can be improved, and their application in health food dosage forms can be further broadened.

## 5. Main Health Benefits of Protein-Based Ingredients

Based on the aforementioned statistics, the top four health functions, namely, boosting immunity, improving bone density, improving skin hydration, and relieving physical fatigue, are highlighted.

### 5.1. Helps Boost Immunity

**Figure 6:**
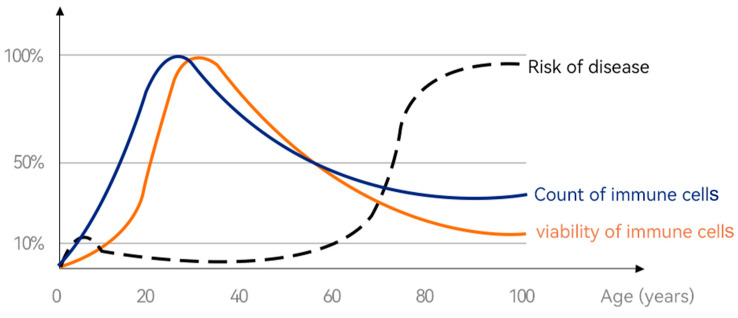
Changes in immune cell viability from “birth” to “lifetime”. (Immune cells’ senescence causes the body to age, and the key to anti-ageing lies in the vitality and number of immune cells. Figure courtesy of [22]).

The means of treating rabies by applying the brain tissue of a rabid dog to the bite wound in the “Elbow Hou Bie Qiang Fang” is considered to be the germ of immunization thinking in China. The three main stages of immunity are the recognition stage (distinguishing between self and foreign pathogens), the activation stage (activating immune cells and immune molecules to produce immune responses), and the effector stage (removing pathogens and other harmful substances through multiple immune effector mechanisms), producing antigens or induce immune tolerance to maintain the health of the body and the stability of the internal environment. The need for immunity in humans continues throughout the lifespan, and Figure 6 illustrates immune cell viability, immune cell counts, and risk of disease throughout a human lifetime. In general, infants have poor immunity from 0 to 6 months of age, after which the immune system begins to gradually improve [19,20], and mothers are exposed to foods during pregnancy, which affects the mother’s intestinal flora and further determines the adaptive immunity of the newborn [21].

The modulatory and promoting effects of proteins and peptides on immunity have been studied. At present, there are 371 protein products used in China’s health food to help enhance immunity. The raw materials that are frequently used are soy protein isolate, whey protein, whey protein concentrate, soy protein, collagen, soy peptide, and lactoferrin.

Commercially available plant proteins contain three types: protein powders, which contain 10 to 20 percent protein; protein concentrates, which contain 55 to 60 percent protein; and isolates, which contain more than 80 percent protein [23]. As a result, soybean isolate proteins with high protein content have been used most frequently in immune-enhancing health foods. Soybean is a high-quality source of protein, consisting of about 40% protein and 20% oil (dry weight) [23], and contains various active peptides, isoflavones, and saponins. Soy protein has a similar amino acid composition to animal proteins compared to other plant proteins [24]. Earlier studies found that casein and soy protein significantly increased thymus weight and cellular composition in the immune organs of immunocompromised mice [25,26]. Further hydrolysis of soy protein can obtain soy peptides, which are easily absorbed by the body and can promote the proliferation and cytosolic activity of macrophages, while soy peptides can also induce the expression of iNOS mRNA to promote the production of NO and can promote the mRNA expression level of IL-6 and TNF-α [27], of which NO plays an important role as a signaling molecule to regulate the T-cell-mediated immune process [28,29], while IL-6 and TNF-α are very important contributors to host defense. Soy peptides have also been found to have better activity in modulating the cellular immune system in clinical studies [30]. Soybeans can be deoiled at low temperatures to obtain soy protein isolate (SPI), which has a higher content of proteins compared to soy protein, and it is a mixture of several proteins formed, with β-glucanoglobulin (7S globulin) and dextranoglobulin (11S globulin) as its main components [31].

Among them, 7S globulin is a glycoprotein formed by the combination of polysaccharide and N-terminal aspartic acid, and 11S globulin is a non-glycoprotein formed by the accumulation of acid-alkaline subunits through hydrogen bonding and hydrophobic interaction, which play a decisive role in the functional properties of soybean protein isolate [32,33,34]. At the same time, in cellular immunity, fermented soybean protein peptides have also attracted much attention in recent years. Fermented soybean protein peptide can significantly improve the proliferation ability of spleen lymphocytes, T-cells, and B lymphocytes in mice and increase the level of serum hemolysin in mice, so it also has a certain regulatory effect on humoral immunity. In non-specific immunity, fermented soy protein peptides can also enhance the killing power of natural killer cells (NK) [35]. Whey protein contains protein content greater than or equal to 25%, which is a by-product of the production of cheese or casein and mainly contains a variety of active ingredients such as β-lactoglobulin, α-lactalbumin, immunoglobulins, growth factors, lactoferrin, etc. The ratio of essential amino acids (including branched-chain amino acids, which contain about 26%) is similar to that required by the human body [36,37], and whey proteins contain a variety of active peptides, which are enzymatically cleaved and released, which further promotes the health needs of the organism [38]. Dietary whey protein enhances the humoral immune response and cell-mediated immune response to sheep erythrocytes [39]. In the process of obtaining lactose from whey via ultrafiltration or nanofiltration techniques, the whey protein retained by the ultrafiltration membrane is usually recovered and simultaneously concentrated to produce whey protein concentrates, which are up to 80% protein [40] and are lactose-free, making them suitable for use in lactose intolerant populations. In addition to the above pathways, β-lactoglobulin trypsin separated via membrane filtration can exert immunomodulatory effects by inducing the secretion of transforming growth factor-β and regulating T-cell differentiation because whey contains many immunomodulatory peptides that occur naturally or serve as whey protein primary sequences, which can be released during intestinal digestion and can also be generated by in vitro enzymatic hydrolysis [41]. Numerous studies have demonstrated the immune-enhancing effects of whey protein concentrate, as evidenced by its ability to enhance humoral immune responses to a range of heterologous antigens [42] and increase the proliferative response of splenic lymphoid tissues, as well as the phagocytic activity of blood and peritoneal leukocytes in mice [43]. Currently, most of the approved health food products containing protein-based ingredients and claiming to help enhance immunity are formulated with SPI (or soy protein) and whey protein (or whey protein concentrate) as the main formulation of the product. Whey and soy proteins have different digestibility, with whey proteins being hydrolyzed faster and released into the plasma [44] and soy proteins being digested slower [45], which helps to continuously supply the body with nutritional requirements and immune support. In addition to this, some products use collagen (peptides), and it has been shown that collagen peptide intake significantly enhances the thymic index, interleukin-2 (IL-2) secretion, and promotes a significant increase in the CD4+/CD8+ ratio in mice during glucocorticoid-induced immunosuppression. Moreover, collagen peptides were also effective in enhancing some of the immune functions of mice under X-ray irradiation combined with simulated weightlessness, improving the total number of leukocytes and lymphocytes; the total number of splenocytes; and large reductions in T-cells, CD4+ and CD8+ T-cells, B-cells, and natural killer cells in the peripheral blood of the animals. However, reports supporting the enhancement or modulation of immunity related to collagen in studies have been rarely reported, suggesting that the mechanism of action and rationale for the formulation of current health food products approved to contain collagen and claimed to contribute to the enhancement of immunity deserve further discussion and optimization.

At present, most of the formulas of health foods that help to enhance immunity are mainly combined with SPI (or soy protein) + whey protein (or whey protein concentrate), which is a classic “double protein” combination of animal protein + vegetable protein to help provide rich nutrition for subjects, especially a series of active peptides that are further hydrolyzed and released by a variety of proteins in the body that are more conducive to the body’s absorption and access to immune regulation. Delayed allergy (DTH) and the ConA-induced mouse spleen lymphocyte transformation assay (MTT method) were used to evaluate the cellular immune function of mice, and the analysis of experimental data showed that the compound protein powder had the effect of enhancing the proliferation of mouse lymphocytes, suggesting that the compound protein powder in this experiment had the function of enhancing the immunity of mice [35]. At the same time, some products add collagen and plant extracts, but most of them are added as secondary raw materials of the product, so the effect of the addition to help enhance immunity is worthy of further discussion and research, but this also suggests that in addition to the classic combination of animal protein + plant protein, more scientific combinations are urgently needed to meet the more health needs of the population.

### 5.2. Helps Improve Bone Density

**Figure 7:**
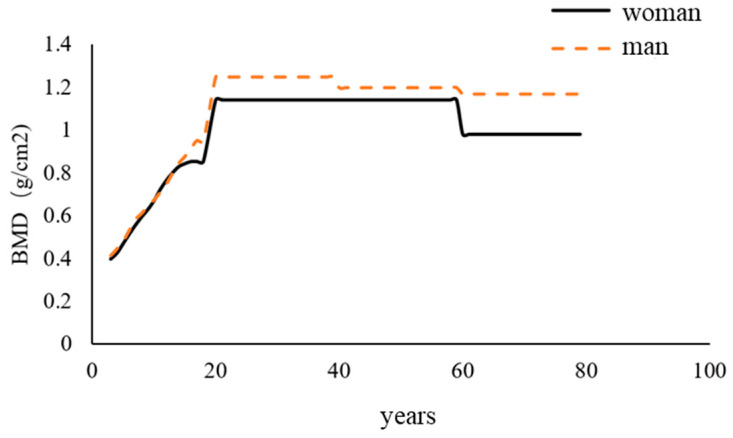
Changes in bone mineral density over a person’s lifetime.

Osteoporosis (OP) results in decreased bone mass and bone strength, which predisposes to the development of fractures and bone fragility, where bone mineral density (BMD) of the hip and/or spine is the hallmark indicator for determining the occurrence of OP [46], and bone strength is equal to the addition of bone mass to BMD. OP is common in the elderly, and the incidence is significantly more than three times higher in women than in men [47]. Figure 7 demonstrates the changes in BMD over a person’s lifetime, with BMD seeing a gradual increase during adolescence [48] and a subsequent gradual decrease in BMD with increasing age, especially in the female population, where the rate of decrease in BMD is much higher than that of males [49]. Bones are mainly composed of bone (including compact bone and cancellous bone), bone marrow (divided into red bone marrow and yellow bone marrow), periosteum, and articular cartilage [50]. Among them, osteoclasts (OC) are mainly responsible for bone resorption and antagonize with osteoblasts, which are responsible for the formation of new bone to maintain bone homeostasis, a process called bone remodeling [51], which belongs to the category of bone metabolism. When there is an increase in osteoclast activity and/or a decrease in osteoblast activity, both result in greater bone resorption than bone formation, which leads to structural disruption of cancellous bone, a decrease in bone strength and, ultimately, can induce OP [50,52]. Currently, protein-based ingredients applied to increase BMD are mainly formulated through a pathway that promotes bone remodeling balance and calcium absorption.

Currently, there are a total of 207 products containing protein-based ingredients for improving bone density and health functions, of which only five products use plant proteins (two soy protein powders and three soy protein isolates). This may be because animal proteins are more readily absorbed by the body compared to plant proteins [53,54], and animal proteins not only have a complete amino acid profile but also usually contain a certain level of calcium, vitamin D, and/or vitamin B_12_, nutrients that are closely related to the improvement in bone density [55]. This also suggests the need to further explore the quantitative relationship and mechanism of action of dietary proteins in synergizing with other nutrients, such as calcium, vitamin D, and B vitamins, to promote bone mineral density in the course of research on the use of protein-based ingredients in formulations to increase the function of BMD. Currently, the more classical health food formulation pairings applied to increase BMD function are casein phosphopeptides (CPP) + calcium (Ca) or supplemented with other ingredients such as magnesium (Mg) and/or Vitamin D_3_. This suggests that the pairing of CPP with Ca and Mg may play a role in increasing BMD by regulating the balance of bone remodeling from multiple pathways, such as promoting bone formation, enhancing Ca and Mg resorption, and decreasing bone resorption. Vitamin D3 supplementation has been shown in numerous studies to have a protective effect on BMD in the lumbar spine, femoral neck, and total hip [56,57], and, as such, it is commonly used in the dispensing of health foods to improve BMD.

In addition, CPP+ collagen-based raw materials (mainly bone collagen, collagen, hydrolyzed collagen, marine fish collagen oligopeptide, and freshwater fish collagen peptide) also have more applications. One study found that the oral administration of collagen peptides had a significant improvement effect on bone metabolism in calcium-deficient rats, restoring BMD in the femur and lumbar spine of calcium-deficient rats [58]. Fish skin collagen hydrolysate restored low BMD and reduced bone loss in femoral and tibial tissues of ovariectomized rats [59]. Moreover, collagen-based ingredients also help to improve arthritis [60,61], and they can fulfill a wide range of bone health needs of the target population. In a clinical study, the elderly diagnosed with mild arthritis and sarcopenia were divided into a supplement intervention group (PR) and a placebo control group (PL), and the subjects were given a 15 g protein/peptide nutritional supplement or placebo once in the morning and evening for 12 weeks. The study found that cytokines (CK), creatine kinase isoenzyme (CKMB), growth promotion factor (IGF-1), and serum albumin were significantly increased in the PR group, while C-reactive protein (CRP) was significantly reduced. Thus, the supplement improved the symptoms of elderly patients with osteoarthritis [62].

At present, the functional evaluation of health food products that help to improve bone density only requires animal experiment evaluation, and animal models can be divided into two categories: calcium supplementation and the promotion of bone metabolism. The mechanism of action of most formulations is mainly to promote the balance of bone remodeling and calcium absorption, and some formulations also add ingredients that help improve osteoarthritis, which meets the bone health needs of the target population.

### 5.3. Helps Improve Skin Hydration

**Figure 8:**
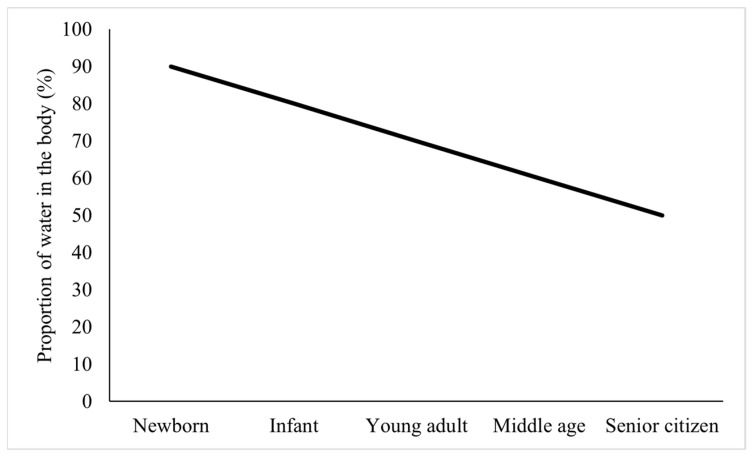
Changes in water in the body over the course of a person’s lifetime.

The functional evaluation method of health food that helps to improve the skin moisture condition was only carried out to evaluate human trial food experiments, and the evaluation method involved a clean cotton ball dipped in distilled water to clean the tested part of the subject in a quiet state. It was then dried for 15 min to measure moisture. At present, a total of 79 health food products containing protein-based raw materials to help improve skin moisture have been approved, mostly using collagen, fish collagen, marine fish skin collagen oligopeptide, and collagen peptide as the raw material of the product. The product audience is also mostly oriented to women. Studies have shown that the moisture in the skin of animals decreases with age [63], and Figure 8 demonstrates the change in moisture in the organism during a person’s lifetime [64].

The skin is the first line of defense of the immune barrier and the main physical barrier against excessive water loss. It is composed of the epidermis, the dermis, and the subcutaneous tissue in order from the outside to the inside, and with aging, as well as the influence of the external environment, the water in the skin decreases. Collagen and hyaluronic acid are important components of the skin necessary for maintaining skin elasticity and hydration [65,66]. The oral administration of collagen peptides promotes the synthesis of collagen and hyaluronic acid [67]. Studies have confirmed that tablets made with collagen peptides as the main ingredient significantly improved skin moisture in subjects [68]. Powder formulated with collagen and soy peptide as the main raw materials was judged to have the health care function of improving skin moisture and was safe and non-toxic, as confirmed by human trial food evaluation [69]. However, these studies did not provide an in-depth study and discussion of the mechanisms by which protein-based ingredients improve the skin hydration status. Currently, studies on improving skin hydration lack animal experimental evaluation models, so the physiological mechanisms for improving the skin hydration status are unclear. However, some studies have found that aquaporins (AQP) have an important role in water transport and hydration in the human skin epidermis [70]. In 2003, Peter Agre won the Nobel Prize for his discovery of AQP [71]. AQP is a family of transmembrane channel proteins that can rapidly transport water molecules, and its isoform AQP3 is abundantly expressed in the epidermis and dermis. It was found that the dermal water content in the skin of 20-month-old aged mice was significantly lower than that of 3-month-old young mice, accompanied by a significant decrease in the mRNA expression of AQP3 [72], which suggests that it may be helpful to improve the skin water status by increasing the expression of AQP3. It has been found that the treatment of human HaCaT keratinocytes with collagen hydrolysate extracted from Pangasius hypophthalmus fish skin can improve the decrease in AQP3 expression induced by UV-B exposure, and further animal model evaluation found that it may alleviate the photoaging of deep skin wrinkles, epidermal thickening, and skin moisture loss by inhibiting collagen destruction and epidermal barrier damage. These findings suggest that aquaporins should be of interest in studying skin actinic mechanisms in future studies [73].

### 5.4. Relief of Physical Fatigue

Physical fatigue is the sensation of decreased work capacity and physical functioning caused by physical labor and/or exercise [67]. Studies have shown that strenuous exercise not only leads to loss of peripheral muscle capacity but also leads to changes in central nervous system function and induces a variety of diseases [74]. The data show that the number of approved health food products containing protein-based raw materials that claim to have the function of relieving physical fatigue totaled 50. The main raw materials used are whey protein, whey protein concentrate, SPI, soy peptide, soy protein, and more with taurine to match, followed by raw materials such as wolfberry and western ginseng.

Protein-based ingredients contain anti-fatigue peptides, such as sea cucumber peptides [75], pea polypeptides [76], and soy protein peptides [77], which promote glycogen synthesis, eliminate metabolic by-products, increase the level of antioxidant enzymes in the body, inhibit inflammation, and regulate neurotransmitters, among other pathways to improve physical fatigue [73]. Studies have shown that shrimp head autolyzed active peptides have a significant effect on anti-fatigue in mice, and shrimp head autolyzed active peptides can reduce the oxidative stress response, improve exercise ability, and exert anti-fatigue effects in mice by activating the Keap1/Nrf2/ARE signaling pathway in mice [78]. The results of in vitro antioxidant and animal behavior studies have shown that band fish glycoprotein (HGP) has a good free radical scavenging capacity and can improve exercise and mental fatigue in BALB/c mice, with anti-fatigue efficacy [79].

Each protein ingredient has unique advantages in relieving physical fatigue and can synergize to play a more significant role through a reasonable combination. Whey protein is rich in tryptophan [24], while soy protein is rich in branched-chain amino acids, which can promote muscle growth and recovery [80] and effectively reduce physical fatigue [81,82]. Studies have shown that “soy + whey” dual protein can maintain a high concentration of tryptophan and branched-chain amino acids sustained release [83]; by supplementing the combination of soy protein and whey protein for 7 weeks, it can significantly prolong the swimming time of fatigue model rats. After ingesting this formula for 60 min, the plasma leucine, isoleucine, and valine levels were significantly higher than those of the whey protein single formula group and the blank control group; there were increased serum lactate dehydrogenase and SOD activities and reduced serum MDA levels, indicating that the ingestion of dual protein after resistance exercise can be significantly higher than the serum MDA levels. Valine levels were significantly higher than those of the whey single-component protein group and the blank control group; increased serum lactate dehydrogenase and SOD activities and decreased serum MDA levels were noted, suggesting that the ingestion of dual proteins after resistance exercise can improve exercise performance and ameliorate exercise-induced fatigue in rats [84]. In addition to animal and plant protein pairings, the pairing of protein-based raw materials with plant extracts has also been shown to have better anti-fatigue effects. Hippocampus hydrolysate showed significant anti-fatigue activity when paired with red ginseng [85]. Oyster peptide paired with ginseng extract prolonged the forced swimming time in mice; decreased serum lactate, blood urea nitrogen, and lactate dehydrogenase activity; and elevated skeletal muscle liver glycogen content and antioxidant capacity [86]. Therefore, it is a necessary means to maximize the efficacy of healthy food through appropriate compounding, and these findings need to be supported by a large number of basic and mechanistic research studies.

## 6. Recommendations for Future Research

### 6.1. Increase in Research on the Formulation of Protein-Based Raw Materials

Raw protein materials are rich in variety and high in nutritional value, and they are digested by the body to obtain a series of proteins, active peptides, amino acids, and other active ingredients, thus providing the body with a variety of health support. Currently used in health food, raw protein materials based on use can be divided into five categories: health food ingredients, food nutrient fortification, health food supplements, new food ingredients, and general food ingredients. In this paper, through data statistics and analysis, it was found that the main raw protein materials applied in health food are mostly CPP, SPI, whey protein, collagen, spirulina, whey protein concentrate, soy protein, marine fish skin collagen oligopeptide, soy peptide, hydrolyzed collagen, collagen, lactoferrin, fish collagen, selenium protein, fish protein, and colostrum alkaline protein. The main health functions applied are used to strengthen immunity, improve bone density, improve skin hydration, and relieve physical fatigue conditions. Among them, to help strengthen the immune system, animal protein + plant protein “double protein” is the most common way; to help improve bone density to CPP + Ca and/or with other divalent minerals, plant extracts are mainly used; to help improve the skin moisture status of the product, collagen, fish collagen, marine fish collagen oligopeptide, and collagen peptide are commonly used as a raw material for the product; to relieve physical fatigue, whey protein, whey protein concentrate, SPI, soy peptide, and soy protein are as raw materials in the product formulations, and most of them are used to be used in conjunction with taurine, followed by goji berry and Panax quinquefolius. The formulae used in these functions are very classic, but at the same time, the result also reflects that the formulae of similar products are less differentiated, which suggests that future research on the raw protein materials and other raw materials, such as plant extracts and micronutrients, should be increased. Specifically, this includes research on the mechanism of the synergistic effect of formulations and research on the relationship between quantity and effect. Secondly, the excavation and compliance declaration of new protein-based raw materials should be increased to improve their application value.

### 6.2. Enhanced Delivery System Study of Protein-Based Raw Materials

Data show that the main dosage form of protein-based raw materials in health food is solid preparation, which is closely related to the nature of these materials. It is suggested that the study on the delivery system of protein-based raw materials in future research be boosted to solve the problems of poor flavor, stability, and bioavailability, thus improving the choice of dosage forms and health care functions of protein-based health food. However, the materials of the preparation system of the delivery system may have some potential toxicity, so the research should be combined with toxicological studies to ensure the completeness of the product. At the same time, the encapsulated raw protein materials processing technology is complex, which will inevitably bring about an increase in cost. Thus, the research process should be combined with the actual situation to create raw protein materials with efficient and economical encapsulation technology to further the realization of raw materials to provide assistance for large-scale production.

### 6.3. Strengthen the Research of Raw Protein Material-Based Preparations

Raw protein materials occupy a key position in the global health food field. As consumers become more health-conscious, there is a growing demand for products that can provide high-quality protein supplements and have specific health benefits. Therefore, it is necessary to strengthen the research on raw protein materials-based preparations. First, some raw protein materials (such as plant proteins) may have special smells and tastes, which affect consumer acceptance to a certain extent. Therefore, in the process of preparation, these undesirable flavors should be effectively masked or improved so as to improve their market acceptance. Second, as we all know, protein is a relatively unstable biological macromolecule, which is susceptible to changes in environmental conditions such as temperature, humidity, and pH during processing, storage, and transportation, resulting in protein denaturation, aggregation, or degradation, thereby reducing its activity and nutritional value. Therefore, it is also important to conduct a stability study of the formulation process. Formulation optimization and the screening of the combination of synergistic and stable raw materials can extend the shelf life of products. Third, the bioavailability of protein is an important factor in determining its health effect. Therefore, it should also be fully considered in the process of preparation. In terms of preparation technology, we can consider using microencapsulation technology, nanotechnology, liposome technology, and other methods to improve the stability of raw materials and further improve the bioavailability of products so as to support their health care effects.

## 7. Summary

In short, through data analysis, this review found that during 1996~2024, a total of 1142 health foods containing raw protein materials were approved in China, which can mainly be divided into animal protein, plant protein, microbial protein, and raw peptide materials, and raw peptide materials comprise the majority. The compliance applications of these ingredients are mainly related to China’s five categories of food regulations. These health foods involve raw protein materials: in terms of dosage form, they mainly include solid preparations, and in terms of functional claims, they mainly help to enhance immunity, help improve bone density, help improve skin moisture status, and relieve physical fatigue. In the application of raw materials, it was found that the number of products used in raw materials, such as casein phosphopeptide, soybean protein isolate, whey protein, collagen, spirulina, and other raw materials, was relatively high.

In this study, the data on health foods containing raw protein materials are sorted out and analyzed in detail, and the application of raw protein materials in Chinese health foods is comprehensively introduced. The four functions of enhancing immunity, improving bone density, improving skin moisture status, and relieving physical fatigue are discussed in detail. However, this study focuses on health foods in China and does not discuss and analyze protein ingredients in detail in health foods around the world, nor do we explain policies, so we will continue to improve these deficiencies in the future.

## Figures and Tables

**Table 1:** Basis of use and details of healthful raw protein food materials.

Classification	Raw Materials	Basis of Use
Health Food Raw Materials	Selenoprotein	Health Food Ingredients Catalog No. 22 of 2023 Nutrient Supplements (2023 Edition)
	Casein phosphopeptide + calcium	
	Soybean isolate	Health Food Ingredients Catalog Soybean—Isolated Protein
	Whey protein	Health Food Ingredients Catalog—Whey Protein
	Spirulina	Catalog of Health Food Ingredients—Spirulina
Food Nutrition Reinforcement	Casein phosphopeptide (CASP)	GB 31617-2014 National Standard for Food Safety Food Nutritional Enrichment—Casein Phosphopeptide
	Lactoferrin	GB 1903.17-2016 National Standard for Food Safety Food Nutritional Fortification—Lactoferrin
	Selenoprotein	GB 1903.28-2018 National Standard for Food Safety Food Nutritional Fortification—Selenoprotein
Health Food Accessories	Sodium caseinate (chemistry)	GB 1886.212 National Standard for Food Safety Food Additives —Sodium caseinate (also known as sodium caseinate)
	Ice-structured protein	Ice-structured protein
New Food Raw Material	Hydrolyzed egg yolk powder	Former Ministry of Health Bulletin No. 20 of 2008
	Colostrum alkaline protein	Former Ministry of Health Bulletin No. 12 of 2009
	Milk Alkaline Protein	Former Ministry of Health Bulletin No. 18 of 2009
	*Lumbricus* protein	Former Ministry of Health Bulletin No. 18 of 2009
	*Chlorella pyrenoidosa*	Former Ministry of Health Bulletin No. 19 of 2012
	*Euglena gracilis*	Former Ministry of Health No. 10 of 2013
	*Nostoc sphaeroides*	Health Commission No. 10 of 2018
	*Nannochloropsis gaditana*	Health Commission No. 5 of 2021
	*Chlamydomonas reinhardtii*	Health Commission No. 2 of 2022
	Yeast protein	Health Commission No. 10 of 2023
General Food Ingredients	Bead peptide powder	Former Ministry of Health Bulletin No. 20 of 2008
	Corn oligopeptide	Former Ministry of Health Bulletin No. 15 of 2010
	Wheat oligopeptide	Former Ministry of Health Bulletin No. 16 of 2012
	The substance is made from edible animal or plant protein as raw material, enzymatically digested by the enzyme preparation for food use permitted by the Standard for the Use of Food Additives (GB2760-2011).	Former Health and Family Planning Commission Announcement No. 3 of 2013
	Potassium caseinate (calcium, magnesium, and sodium)	Announcement No. 897 of the State Health Office Food Letter [2014]
	Milk protein powder	National Health Food Labeling Letter [2016] No. 8 Announcement
	Enzymatic collagen (collagen)	Wei Food New Notice [2011] No. 0003
	…	…
	Star Oil Vine Protein Powder | My Vine Fruit Protein	New Food Ingredients Terminated for Review and Determined to be Common Food Ingredients
	Non-denatured type II collagen/	
	Cartilage Powder with Type II Collagen	
	Milk protein concentrate	
	Elastin (also renamed bonito elastin peptide)	
	Chicken Imidazole Dipeptide Powder (later renamed Chicken Concentrate Powder)	
	Enzymatic Bone Meal	

**Table 2:** Application of protein-based raw materials in health foods.

Classification	Raw Materials
Peptides	Peptides (26 kinds): casein phosphopeptide, marine fish skin collagen oligopeptide, soy peptide, whey protein peptide, marine fish bone collagen oligopeptide, collagen peptide, marine fish oligopeptide, soybean oligopeptide, fish collagen peptide… Hydrolyzed peptides (18 kinds): hydrolyzed collagen, sheep placenta hydrolysate, hydrolyzed proteins… Enzymatic peptides (12 kinds): enzymatic bone calcium powder, enzymatic ovalbumin…
Animal proteins	Whey protein, collagen, whey protein concentrate, collagen, lactoferrin, fish collagen, fish protein, colostrum alkaline protein, casein, ovalbumin, egg white protein …
Plant proteins	Soybean isolate protein, soy protein, protein nucleus chlorella, pea protein, wheat protein, vegetable protein, barley protein, gluten, soy protein fiber, selenium protein
Microbial protein	Spirulina

Note: The ingredients in the table are only those that appear more frequently in approved products.

## Data Availability

No new data were created or analyzed in this study. Data sharing is not applicable to this article.
